# Drug-Induced Hypothyroidism during Anti-Tuberculosis Treatment of Multidrug-Resistant Tuberculosis: Notes from the Field

**DOI:** 10.4236/jtr.2016.43013

**Published:** 2016-08-23

**Authors:** Somashekar Munivenkatappa, Singarajipura Anil, Balaji Naik, Tyson Volkmann, Karuna D. Sagili, Jayachamarajapura S. Akshatha, Shashidhar Buggi, Manchenahalli A. Sharada, Sudhendra Kulkarni, Vineet K. Chadha, Patrick K. Moonan

**Affiliations:** 1 Drug Resistant Treatment Centre, Bengaluru, India; 2 Sri Devarao Shivaram, Tuberculosis and Rajiv Gandhi Institute of Chest Diseases, Bengaluru, India; 3 Revised National Tuberculosis Programme-State of Karnataka, Bengaluru, India; 4 World Health Organization, India Country Office, New Delhi, India; 5 U.S. Centers for Disease Control and Prevention, Atlanta, GA, USA; 6 International Union against Tuberculosis and Lung Disease (The Union), South-East Asia Regional Office, New Delhi, India; 7 National Tuberculosis Institute, Bengaluru, India

**Keywords:** Hypothyroidism, Multidrug Resistance, Tuberculosis, Thyroid-Stimulating Hormone, India

## Abstract

We followed 188 euthyroidic persons undergoing treatment for multidrug resistant tuberculosis (MDR-TB) in the state of Karnataka, India to determine the incidence of hypothyroidism during anti-tuberculosis treatment. Overall, among MDR-TB patients with valid thyroid stimulating hormone (TSH) values, about 23% developed hypothyroidism (TSH value ≥10 mIU/ml) during anti-tuberculosis treatment; the majority (74%) occurring after 3 months of treatment. Among 133 patients who received a regimen that contained ethionamide, 42 (32%) developed hypothyroidism. Among 17 patients that received a regimen that contained para-aminosalicylate sodium, 6 (35%) developed hypothyroidism. Among 9 HIV positive patients on anti-retroviral treatment, 4 (44%) developed hypothyroidism. These results differ from previously reported 4% incidence of hypothyroidism amongst patients who passively reported thyroidal symptoms during treatment, suggesting routine serologic monitoring of TSH throughout the course of treatment for MDR-TB is warranted.

## 1. Introduction

Multidrug-resistant tuberculosis (MDR-TB) is defined as tuberculosis (TB) that is resistant to isoniazid and rifampicin, the two most efficacious anti-TB drugs [1]. MDR-TB has emerged as a major threat to TB control in India with approximately 62,000 new cases occurring annually [1] [2]. In Karnataka state, India, Programmatic Management of Drug-resistant TB (PMDT) started in 2011. All patients with laboratory-confirmed drug-resistant TB are treated with a standard regimen; this includes an intensive phase consisting of kanamycin, cycloserine, levofloxacin, ethionamide, ethambutol, and pyrazinamide for 6 – 9 months followed by a continuation phase of 18 months with cycloserine, levofloxacin, ethionamide, and ethambutol [2]. In the event of drug intolerance or severe adverse reactions to kanamycin and cycloserine, paraaminosalicylate sodium (PAS) may be used as a substitute drug [3]. PAS and ethionamide are known to cause hypothyroidism [4]. Anti-retroviral drugs (stavudine, didanosine, and protease inhibitors) used for treatment of HIV can also cause hypothyroidism [5]. According to studies carried out elsewhere, 54% – 69% of patients might develop hypothyroidism during treatment for MDR-TB [6]–[8].

Under PMDT, serologic testing for thyroid-stimulating hormone (TSH), a biomarker for thyroid functioning, is conducted only amongst MDR-TB patients who passively report experiencing signs and symptoms of hypothyroidism. However, because hypothyroidism often presents with sub-clinical manifestations that are often masked by other conditions (e.g., arthralgia, depression, ectodermal dysplasia, psychosis, and xeroderma), it may be underdiagnosed [9]. Thus, we conducted a prospective study to find the proportion of cases that develop hypothyroidism during intensive phase of MDR-TB treatment, using routine serological TSH testing.

## 2. Materials and Methods

### 2.1. Patient Selection

All adult (≥15 years of age) patients registered under PMDT in Karnataka during October, 2014-March, 2015 were eligible for enrolment. Those not willing to provide written informed consent or found suffering from hypothyroidism during pre-treatment evaluation were excluded.

### 2.2. Serological Testing

Serologic TSH levels were obtained at baseline, at end of 3 and 6 months of anti-tuberculosis treatment using an ultrasensitive sandwich chemi-luminesent-immunologic assay at a private laboratory in Bangalore accredited by the College of American Pathologist and National Accreditation Board for Testing Calibration. Hypothyroidism was defined as TSH value ≥10 mIU/ml irrespective of clinical symptoms. Patients found suffering from hypothyroidism were provided free treatment with levothyroxine according to international guidelines [9].

### 2.3. Data Collection Data Management and Statistical Analysis

Patient characteristics, anti-tuberculosis drugs prescribed, and TSH values were obtained from the PMDT register, treatment cards and laboratory reports. Data was recorded in a structured data collection sheet, double entered, validated and analysed using EpiData software (EpiData Association, Odense, Denmark).

Proportions developing hypothyroidism and 95% confidence intervals (CI) were calculated. Chi-square was used to detect differences in proportions that developed hypothyroidism (alpha <0.05).

### 2.4. Ethical Considerations

The study was approved by the Institutional Ethics committees of Rajiv Gandhi Institute of TB and Chest Diseases, Bangalore, National Tuberculosis Institute, Bangalore, the Ethics Advisory Group, International Union Against Tuberculosis and Lung Disease (The Union), and the Division of Global HIV and Tuberculosis, Centers for Disease Control and Prevention, Atlanta, GA USA

## 3. Results

Of 194 persons being treated for MDR-TB, 5 (2.5%) patients did not receive a TSH test at baseline, and one was found to be suffering from hypothyroidism during pre-treatment evaluation; these patients were excluded from analysis. Of the remaining 188 patients, 165 (88%) received ethionamide, 5 (3%) received PAS and 18 (10%) both PAS and ethionamide. There were 19 patients who were HIV positive, 9 received ART. Excluding those who died (n = 7), lost to follow-up (n = 12) and transferred out (n = 1) by the third month, 168 were eligible for TSH testing. Of them, 33 were not tested due to for various programmatic reasons. At 3 months, 135 (80%) were tested of which 12 developed hypothyroidism. Excluding those that had developed hypothyroidism by 3 months, 123 were eligible for TSH testing at 6 months. Of them, 8 were not tested. Of 115 tested, 31 additional patients developed hypothyroidism between 3 and 6 months. Thus overall, 43 (23%) developed hypothyroidism during anti-tuberculosis treatment (95% CI: 17% – 29%).

With the exception of females in the first three months of anti-tuberculosis treatment (17% females vs 5% males; p = 0.018), overall risk of developing hypothyroidism was not found related to age, sex or medication received (Table 1). It is worth noting, however, that among 133 patients who received a regimen that contained ethionamide, 42 (32%) developed hypothyroidism. Among 17 patients that received a regimen that contained PAS, 6 (35%) developed hypothyroidism. Also among 9 HIV positive patients on ART, 4 (44%) developed hypothyroidism.

## 4. Discussion

Among MDR-TB patients with valid TSH values, about 23% developed hypothyroidism during antituberculosis treatment, with the majority (74%) occurring after 3 months of treatment. These results differ from a report using routine programmatic monitoring data (TSH conducted only among symptomatic patients) where 4% developed hypothyroidism [10]. These results suggest that serological TSH monitoring may be important throughout the course of MDR-TB treatment. All patients should receive serological TSH monitoring during MDR-TB treatment to detect subclinical cases of hypothyroidism, which if not treated may lead to poor quality of life, and unfavourable outcomes including death and loss to follow-up. Findings of this study could have application to all patients treated under PMDT in India. A limitation of the present study was that TSH testing was performed only at the third and sixth months of treatment. It is likely that testing at more regular intervals and throughout the entire length of MDR-TB treatment would increase the time to detection of hypothyroidism. Future studies are needed to find out the proportion of MDR-TB patients developing hypothyroidism during continuous phase as well, as susceptibility for hypothyroidism may continue beyond IP. A substantial number of patients (11%) either died or were lost to follow-up. It is possible that some these patients may have suffered from hypothyroidism, thus underestimating incidence.

## 5. Conclusion

Undiagnosed hypothyroidism has serious potential sequelae to MDR-TB patients and increases the risk for additional physical and mental health problems. The current PMDT policy of waiting for thyroidal symptoms to develop may underestimate the true burden and put patients at unnecessary risk and worsen clinical outcomes. Routine TSH testing should be considered for all patients receiving anti-tuberculosis treatment for MDR tuberculosis, especially those receiving regimens containing PAS and ethionamide or antiretrovirals.

## Figures and Tables

**Table 1:** Demographic and treatment characteristics of patients receiving antituberculosis drug treatment for multidrug-resistant tuberculosis by thyroid stimulating hormone testing—Karnataka, India, October 2014 through January 2015.

	At the end of 3rd Month						At the end of 6th month					

	TSH tested	TSH ≥10 mIU/ml (hypothriodism)		TSH ≤9 mIU/ml (normal)		p-value	TSH tested	TSH ≥10 mIU/ml (hypothriodism)		TSH ≤9 mIU/ml (normal)		p-value

	n = 135	n = 12 (8.9%)		n = 123 (91.1%)			n = 115	n = 30 (26.1%)		n = 85 (73.9%)		
**Characteristic**												
**Age (in years)**						0.65						0.74
**<15**	1	0	0.0%	1	100.0%		1	0	0.0%	1	100.0%	
15 –24	21	4	19.0%	17	81.0%		17	4	23.5%	13	76.5%	
25 –34	36	2	5.6%	34	94.4%		31	7	22.6%	24	77.4%	
35 –44	33	3	9.1%	30	90.9%		31	10	32.3%	21	67.7%	
45 –54	19	2	10.5%	17	89.5%		18	6	33.3%	12	66.7%	
55 –64	21	1	4.8%	20	95.2%		13	2	15.4%	11	84.6%	
>65	4	0	0.0%	4	100.0%		4	2	50.0%	2	50.0%	
**Sex**												
Female	48	8	16.7%	40	83.3%	**0.02**	38	7	18.4%	31	81.6%	0.15
Male	87	4	4.6%	83	95.4%		77	24	31.2%	53	68.8%	
**Medication Received**												
Ethiomide (ETH)	118	11	9.3%	107	90.7%	0.64	99	26	26.3%	73	73.7%	0.68
Para-amino salicylate sodium (PAS)	2	0	0.0%	2	100.0%	- -	2	1	50.0%	1	50.0%	0.97
Both PAS and ETH	15	1	6.7%	14	93.3%	0.75	14	4	28.6%	10	71.4%	0.75
Antiretroviral drugs	19	2	10.5%	17	89.5%	0.79	19	9	47.4%	10	52.6%	0.72

TSH = thyroid stimulating hormone measured at mirco-international units per millilitre.

p-value for Pearson’s chi-square
